# Gut Microbiota, Inflammatory Bowel Disease and the Prioritization of Host Effector Genes: A Two-Sample Mendelian Randomization Study with Independent Replication, cis-eQTL Colocalization and Single-Cell Localization

**DOI:** 10.3390/ijms27198718

**Published:** 2026-09-29

**Authors:** Qiyuan Zou, Jianyi Wang

**Affiliations:** School of Medicine, Guangxi University, No. 100 Daxue East Road, Nanning 530004, China; zouqiyuan7@163.com

**Keywords:** Mendelian randomization, gut microbiota, inflammatory bowel disease, Crohn’s disease, ulcerative colitis, colocalization, cis-eQTL, SENP7

## Abstract

Gut microbial dysbiosis is common in inflammatory bowel disease (IBD), but whether specific taxa are causal, and through which host genes, remains unclear. We integrated two-sample Mendelian randomization (MR), replication, cis-eQTL colocalization, and single-cell mapping to identify microbial taxa with genetically predicted associations and candidate host mediators. Instruments for 211 taxa from MiBioGen (*p* < 1×10^−5^, r^2^ < 0.001) were used in two-sample MR (inverse-variance weighted IVW primary; sensitivity: Egger, weighted median, MR-PRESSO) against IBD, Crohn’s disease (CD), and ulcerative colitis (UC) in the International IBD Genetics Consortium (IIBDGC), with false discovery rate (FDR) correction. Replication was conducted in FinnGen R12; gene prioritization was performed via cis-SMR/coloc.abf using GTEx v8/CAGE eQTLs, and localization in 11,175 colonic cells. MR identified 15 significant associations (FDR < 0.05) across eight features; applying a pre-specified heterogeneity-based rule, 13 across seven independent features were retained as primary associations (strongest NB1n → CD, OR 1.32) and two were downgraded to suggestive; at the independent-signal level, three of 11 associations were replicated and seven were direction-concordant in FinnGen. Colocalization supported shared variants at SENP7 (blood, CD PP.H4 = 0.86, IBD = 0.70) and ZBTB11-AS1 (colon, IBD = 0.76, CD = 0.74); single-cell analysis mapped SENP7 to T/NK, and ZBTB11-AS1/VIPR1 to epithelial/goblet. Replicated MR and colocalization prioritized SENP7 (whole-blood eQTL; minor T/NK-cell detection) and ZBTB11-AS1 (colon epithelium) as candidate effectors, though the latter lacks functional characterization.

## 1. Introduction

Inflammatory bowel disease (IBD), comprising Crohn’s disease (CD) and ulcerative colitis (UC), affects millions worldwide, with rising incidence in newly industrialized regions [1]. IBD reflects an aberrant immune response to commensal microbiota in genetically predisposed individuals [2]. Metagenomic and 16S profiling consistently shows dysbiosis—a reduced abundance of obligate anaerobes (*Faecalibacterium*, *Roseburia*), depletion of butyrate producers, and expansion of pro-inflammatory taxa [2,3,4]. Butyrate and other short-chain fatty acids preserve epithelial barrier function and restrain myeloid cell activation; conversely, microbial products can trigger inflammasome and gasdermin-D-dependent pyroptosis, releasing IL-1β and IL-18 in inflamed mucosa [5,6]. Existing immunomodulators yield incomplete responses in many patients, motivating interest in host–microbe interface interventions. A central bottleneck is identifying which host pathways transduce microbial signals.

Host genetics shape this interface. The International IBD Genetics Consortium (IIBDGC) GWAS has identified >200 IBD susceptibility loci, many in microbial sensing, autophagy and epithelial defence genes [7,8,9]. However, observational microbiome studies cannot distinguish causal microbial drivers from inflammation-induced dysbiosis, because diet, medications and reverse causation confound cross-sectional comparisons. Mendelian randomization (MR) addresses these biases by using germline genetic variants as instrumental variables (IVs) for microbial abundance [10,11].

The MiBioGen meta-analysis (211 taxa, ~18,340 individuals) now enables adequately powered two-sample MR [12]. Several recent MR studies have reported candidate taxa for IBD [13,14,15,16,17], but most lack independent replication—despite evidence that weak instruments and correlated pleiotropy inflate false positives in single-cohort MR [18,19]. Moreover, nearly all stop at taxon-level inference, without connecting microbial IVs to host genes or testing for horizontal pleiotropy via formal colocalization. The biological mechanisms thus remain largely uncharacterized.

Here we performed two-sample MR for 211 gut microbial taxa against IBD, CD and UC in IIBDGC, with independent replication in FinnGen R12. We then integrated cis-eQTL summary-data MR (cis-SMR) and Bayesian colocalization using blood and intestinal eQTLs (GTEx v8, CAGE), and mapped prioritized signals to >11,000 single colonic mucosal cells from health and UC. Our aim was to prioritize candidate host genes through which microbiota-associated variants may influence IBD risk.

## 2. Results

### 2.1. Instrument Construction and Analysis Overview

**Figure 1:**
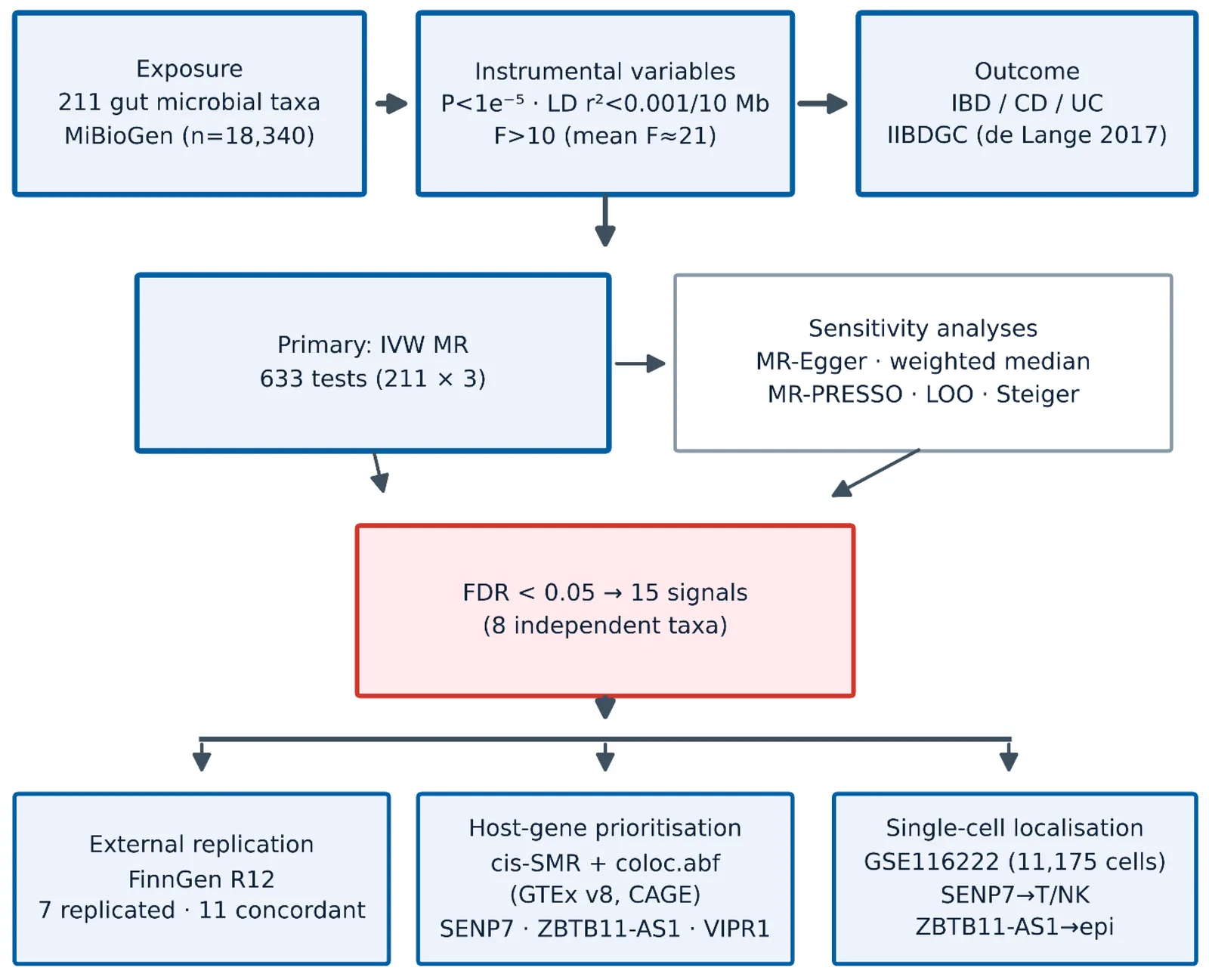
Study design and analysis workflow. A schematic of the two-sample MR pipeline from microbial instrument selection through replication and host-gene prioritization. Genetic instruments for 211 MiBioGen taxa (*p* < 1 × 10^−5^, LD-clumped r^2^ < 0.001/10 Mb, F > 10) were tested against IIBDGC IBD/CD/UC [9] by IVW MR; FDR-significant signals were replicated in FinnGen R12; lead SNPs were followed up by cis-eQTL SMR and coloc.abf in GTEx/CAGE blood and colon tissues; and prioritized genes were localized in GSE116222 colonic single cells. **Abbreviations:** MR, Mendelian randomization; IV, instrumental variable; LD, linkage disequilibrium; IVW, inverse-variance weighted; FDR, false discovery rate; IIBDGC, International IBD Genetics Consortium; eQTL, expression quantitative trait locus; SMR, summary-data-based Mendelian randomization; coloc, Bayesian colocalization; GTEx, Genotype-Tissue Expression; CAGE, Consortium for the Architecture of Gene Expression; SNP, single-nucleotide polymorphism; UC, ulcerative colitis; CD, Crohn’s disease; IBD, inflammatory bowel disease.

**Figure 2:**
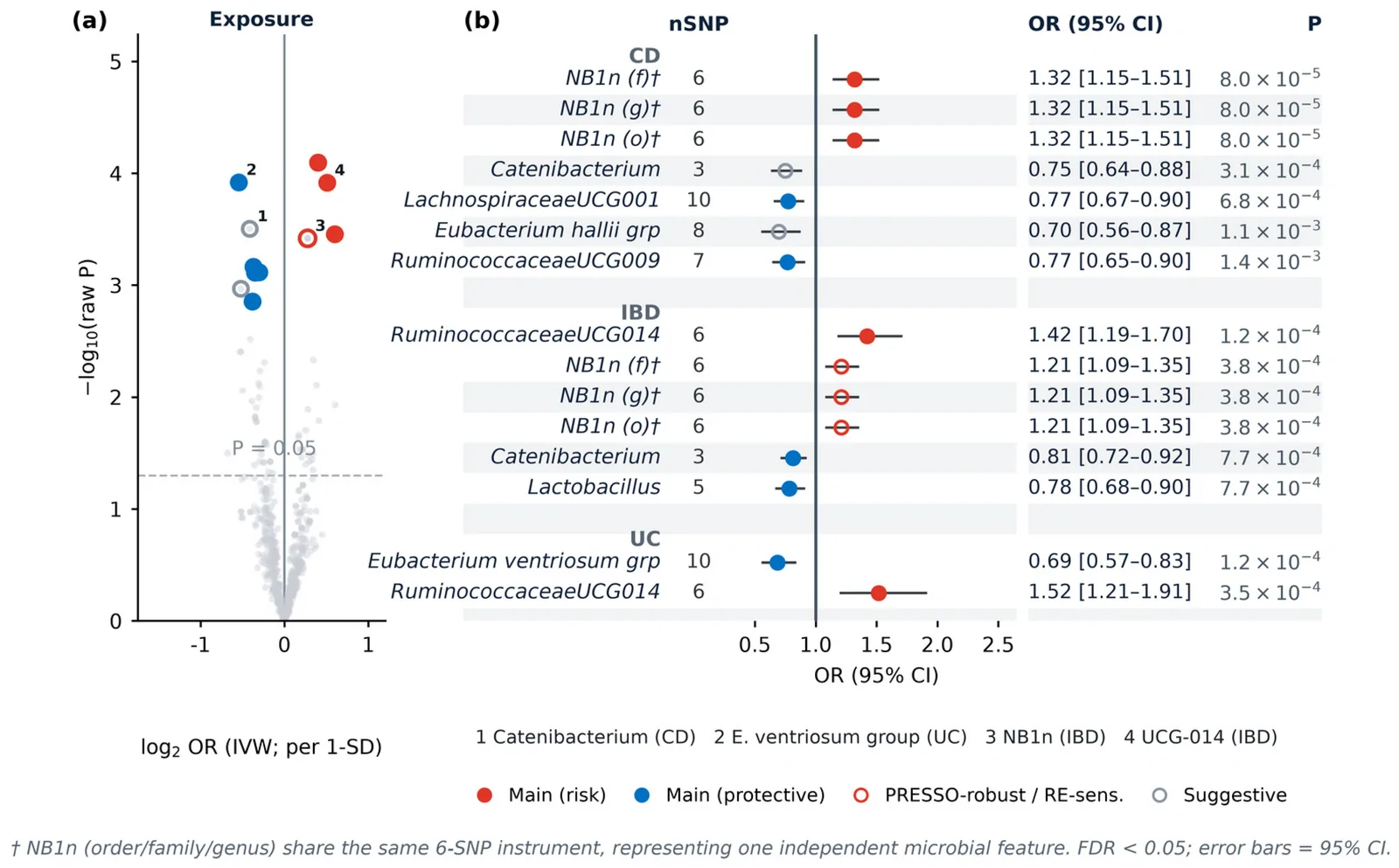
An overview of the MR associations between 211 gut microbial taxa and IBD, CD and UC. (**a**) A volcano-style plot of IVW log-OR against −log_10_(raw *p* value) for all 633 taxon–outcome tests, with the 15 FDR-significant associations highlighted and direction colour-coded (risk, OR > 1; protective, OR < 1); the grey points denote all remaining IVW tests. (**b**) A forest plot of the 15 significant ORs with 95% CIs, with a reference line at OR = 1. The signals are colour-coded by the pre-specified heterogeneity-based classification: Main (solid blue), PRESSO-robust/RE-sensitive (open red) and suggestive (open). Two signals (*E. hallii* → CD and *Catenibacterium* → CD) were downgraded to suggestive after heterogeneity assessment; one (NB1n → IBD) was retained as PRESSO-robust/RE-sensitive. † NB1n (order/family/genus) share the same six-SNP instrument and constitute one independent microbial feature.

For 211 taxa, clumping and strength filtering produced 1278 SNP–trait instruments. Of these taxa, 197 had ≥3 IVs (median F ≈ 21), nine had two IVs and five had one (minimum F ≈ 19.5 across retained instruments). All retained instruments exceeded the conventional weak-instrument threshold (F > 10); the 14 taxa with one or two IVs were assessed by Wald-ratio-type estimates and excluded from multi-SNP pleiotropy diagnostics. A total of 633 taxon–outcome MR tests were performed.Figure 1 shows the overall study design and analysis workflow. Figure 2 provides an overview of the MR associations for all 633 taxon–outcome tests.

### 2.2. Main MR Associations

After per-outcome FDR correction, 15 taxon–outcome associations passed FDR < 0.05, representing eight independent microbial features. Under the pre-specified heterogeneity-based dual-reporting rule, 13 of these associations (across seven independent features) were retained as primary associations; the remaining two (E. hallii → CD and *Catenibacterium* → CD) were downgraded to suggestive (Table 1). Three findings merit emphasis.

First, the strongest signal came from the NB1n order—an unclassified, uncultured *Clostridia* lineage—which increased CD risk (OR 1.32, 95% CI 1.15–1.51; *p* = 8.0 × 10^−5^; FDR = 0.006). This signal appeared consistently at order, family and genus levels, sharing a single six-SNP instrument. The corresponding NB1n effect on IBD (OR 1.21) showed nominally significant heterogeneity and was classified as PRESSO-robust/RE-sensitive: it was retained but flagged because it was not significant under random-effects IVW (*p* = 0.054) yet remained significant after MR-PRESSO outlier correction (corrected *p* = 0.014).

Second, *Ruminococcaceae* UCG-014 increased risk of both IBD (OR 1.42, 1.19–1.70) and UC (OR 1.52, 1.21–1.91).

Third, several taxa showed protective effects: *Catenibacterium* with IBD (OR 0.81); *Lactobacillus* with IBD (OR 0.78); *Lachnospiraceae* UCG-001 with CD (OR 0.77); *Ruminococcaceae* UCG-009 with CD (OR 0.77, q ≈ 0.059); and the *E. ventriosum* group with UC (OR 0.69). The CD-specific protective effects of *Catenibacterium* (OR 0.75) and the *E. hallii* group (OR 0.70) reached FDR significance in discovery but were downgraded to suggestive after the heterogeneity assessment. Under joint correction across all 633 tests, 13 of 15 associated signals remained significant. All effect sizes were modest (OR 0.69–1.60).

### 2.3. Sensitivity Analyses and Replication

Four signals showed nominally significant heterogeneity (Cochran Q *p* < 0.05): *E. hallii* → CD, *Catenibacterium* → CD, *E. ventriosum* → UC and NB1n → IBD. Under the pre-specified dual-reporting rule, the affected signals were re-evaluated with multiplicative random-effects IVW estimates (standard errors inflated by the over-dispersion factor √(Q/df)) and with MR-PRESSO outlier-corrected estimates, and classified accordingly. *E. ventriosum* → UC remained significant under random-effects IVW (*p* = 0.008) and under MR-PRESSO (corrected *p* = 1.7 × 10^−6^) and was retained as a main association. NB1n→IBD was retained but flagged as PRESSO-robust/RE-sensitive because it was not significant under random-effects IVW (*p* = 0.054) yet remained significant after MR-PRESSO outlier removal (corrected *p* = 0.014). *E. hallii* → CD (random-effects *p* = 0.153; MR-PRESSO-corrected *p* = 0.241) and *Catenibacterium* → CD (random-effects *p* = 0.197; MR-PRESSO could not be reliably applied with n = 3 SNPs) were not significant under either estimator and were downgraded to suggestive. MR-Egger intercepts indicated directional pleiotropy for *Catenibacterium* with both CD and IBD (*p* < 1 × 10^−10^). MR-PRESSO flagged global pleiotropy for six of 15 signals. Leave-one-out analyses identified no single SNP that reversed any signal. For the signals retained as robust, the pleiotropy-robust estimators were concordant: MR-Egger and weighted-median estimates matched the IVW direction for NB1n → CD, *Ruminococcaceae UCG-014* → UC, *E. ventriosum* → UC, *Lachnospiraceae UCG-001* → CD and *Lactobacillus* → IBD, whereas both were non-significant for *E. hallii* → CD. The approximated Steiger test supported microbiota → IBD directionality for all 15 signals; given its approximation-based construction, it was used as supportive evidence only.

**Figure 3:**
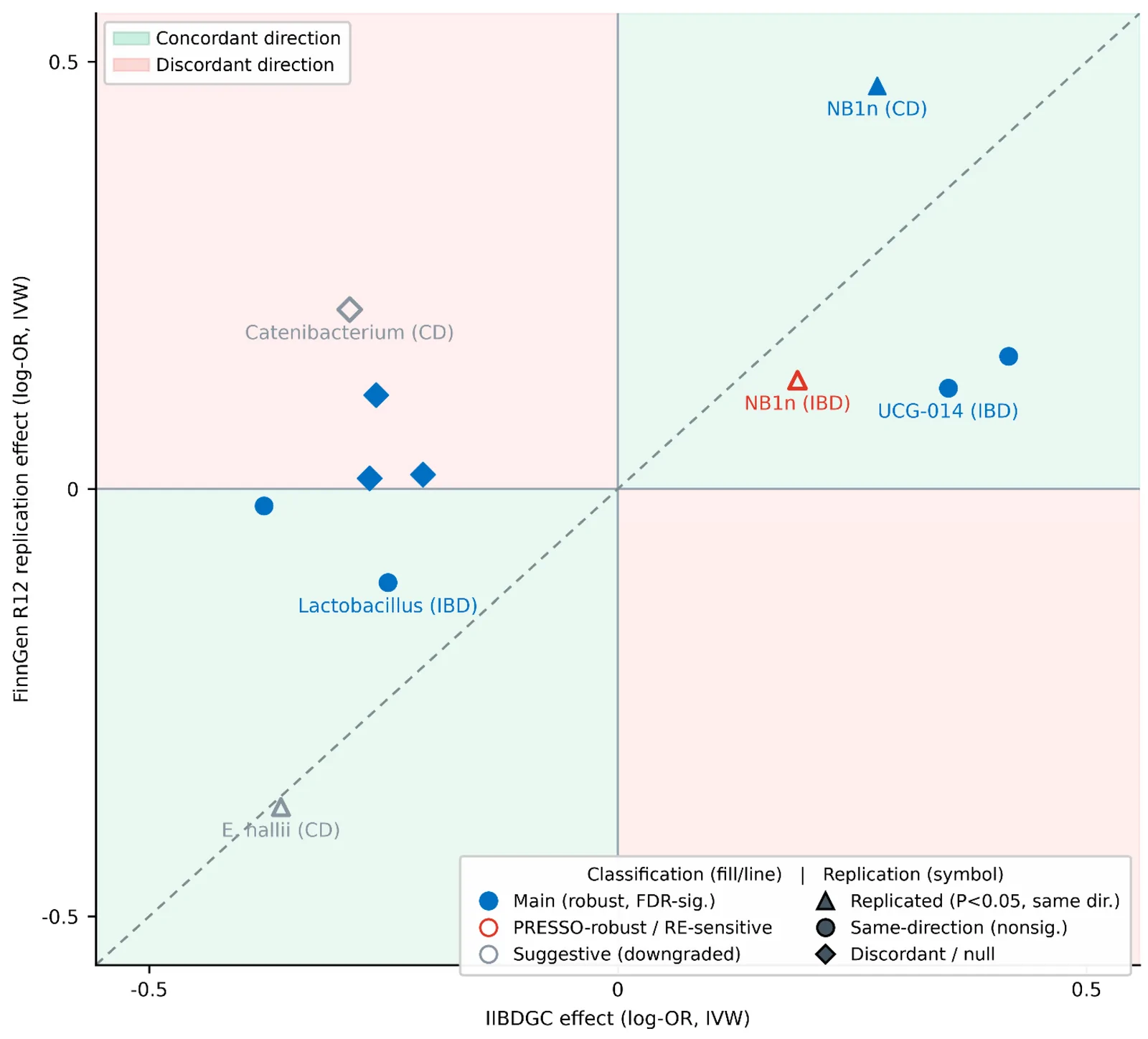
Independent replication of FDR-significant associations in FinnGen R12. A scatter plot of IIBDGC versus FinnGen effect estimates (log-OR) for the 15 FDR-significant signals, shown as 11 unique taxon–outcome associations after collapsing the NB1n lineage (family/genus/order levels share one instrument, as in Table 1); the quadrants indicate concordant or discordant direction. The symbol shape denotes replication status (triangle: replicated, *p* < 0.05 with concordant direction; circle: same-direction but non-significant; diamond: discordant/null); the fill/line denotes classification (solid blue: Main; open red: PRESSO-robust/RE-sensitive; open: suggestive) as in Figure 2. The key signals are labelled.

In *FinnGen* R12, at the independent-signal level, three of 11 unique taxon–outcome associations replicated at *p* < 0.05 with concordant direction: NB1n → CD (OR 1.60, *p* = 2.4 × 10^−4^), NB1n → IBD (OR 1.14, *p* = 0.040) and *E. hallii* → CD (OR 0.69, *p* = 0.018); seven of 11 were direction-concordant. At the nominal test level (counting each of the 15 FDR-significant taxon–outcome associations), seven of 15 (47%) replicated at *p* < 0.05 with concordant direction and 11 of 15 (73%) were direction-concordant; two of the eight independent microbial features had at least one outcome replicate nominally. We emphasize the independent-signal metric throughout, because it avoids over-weighting duplicated taxonomic representations of the same signal, and note that the independent-signal replication rate (three of 11, 27%) is correspondingly more conservative than the nominal tally. *Ruminococcaceae UCG-014* → IBD (OR 1.13, *p* = 0.27) and *Lactobacillus* → IBD (OR 0.90, *p* = 0.071) supported the IIBDGC direction but did not reach nominal significance. *Catenibacterium*, *Lachnospiraceae* UCG-001 and *Ruminococcaceae* UCG-009 showed discordant effects and were treated as non-replicated. Because *FinnGen* contained many fewer CD cases than IIBDGC (2489 versus 12,194), null or reversed CD-restricted estimates with wide confidence intervals were interpreted as inconclusive rather than refutational. Notably, *E. hallii* → CD replicated in FinnGen despite being downgraded to suggestive, consistent with replication being defined independently of heterogeneity classification. Figure 3 shows the IIBDGC–FinnGen replication concordance for the 15 FDR-significant signals.

### 2.4. Gene Prioritization by Colocalization

**Figure 4:**
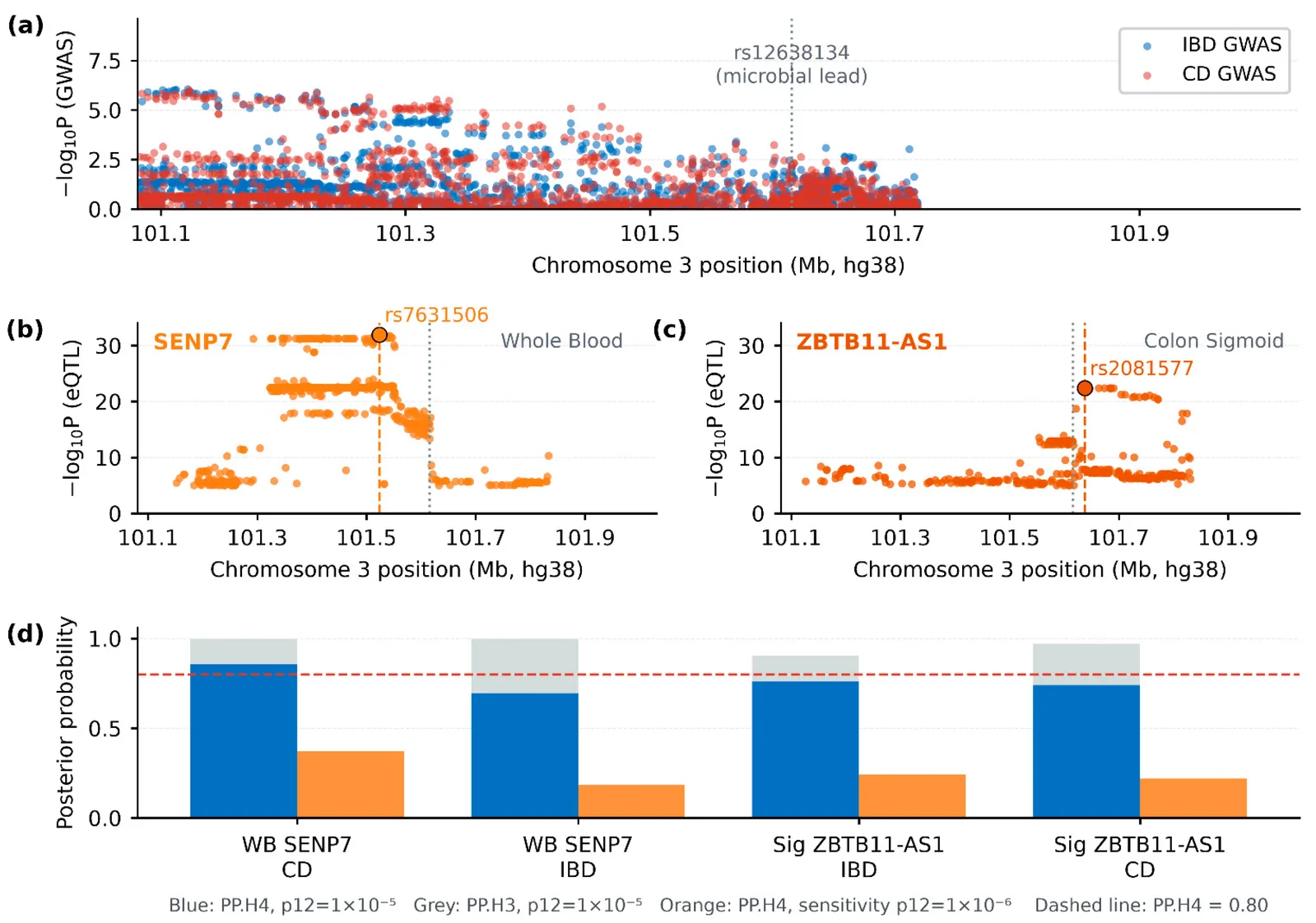
cis-eQTL colocalization and SMR prioritization of the host effector genes. (**a**) Regional association (locus-zoom-style) plots at chr3q12.3, showing IBD and CD GWAS −log10P and the microbial lead SNP rs12638134 (grey dashed vertical line). (**b**,**c**) cis-eQTL −log10P for whole-blood SENP7 (**b**) and sigmoid-colon ZBTB11-AS1 (**c**). (**d**) Bayesian colocalization posterior probabilities at each eQTL–tissue/outcome pair: blue bars, PP.H4 (shared causal variant) under the default prior p12 = 1 × 10−5; grey bars, PP.H3 (distinct causal variants) under the same prior; orange bars, PP.H4 under the stricter sensitivity prior p12 = 1 × 10−6; the dashed line indicates PP.H4 = 0.80. Whole-blood SENP7: PP.H4 = 0.86 (CD), 0.70 (IBD); sigmoid-colon ZBTB11-AS1: PP.H4 = 0.76 (IBD), 0.74 (CD).

Lead microbiota SNPs converged on two loci. For *Ruminococcaceae* UCG-014, lead SNP rs12638134 (chr3q12.3) was a cis-eQTL for SENP7 in whole blood and for ZBTB11-AS1 in sigmoid colon. Colocalization supported a shared whole-blood causal signal at SENP7 for CD (PP.H4 = 0.86) and IBD (PP.H4 = 0.70), but not UC (0.32). In sigmoid colon, by contrast, SENP7 showed distinct causal variants (PP.H3 = 0.97), whereas ZBTB11-AS1 colocalized with IBD (PP.H4 = 0.76) and CD (0.74), but not UC (0.10). cis-SMR Wald ratios were concordant: higher whole-blood SENP7 was associated with lower CD (OR 0.70) and IBD (OR 0.76) risk; higher sigmoid ZBTB11-AS1 was associated with higher CD (OR 1.10) and IBD (OR 1.08) risk. Whole-blood SENP7 effects were replicated in CAGE. The combined cis-eQTL, SMR and colocalization evidence is summarized in Table 2. Figure 4 shows the cis-eQTL colocalization and SMR prioritization of SENP7 and ZBTB11-AS1.

A third locus, *VIPR1*, arose from *Lachnospiraceae* UCG-001’s lead SNP (rs437876), but its upstream taxon association was direction-reversed in FinnGen; *VIPR1* had only 15 SNPs in the colocalization window, yielding no estimable coloc or significant SMR (*p* = 0.38–0.80). It is reported as a biological candidate only.

Under the stricter colocalization prior (p12 = 1 × 10^−6^), whole-blood *SENP7* PP.H4 fell to 0.37 (CD) and 0.19 (IBD); sigmoid *ZBTB11-AS1* PP.H4 fell to 0.24 (IBD) and 0.22 (CD). Gene prioritization therefore rests on the triangulated evidence (cis-SMR, CAGE replication, concordant directions and single-cell localization), not on colocalization alone.

### 2.5. Single-Cell Localization

**Figure 5:**
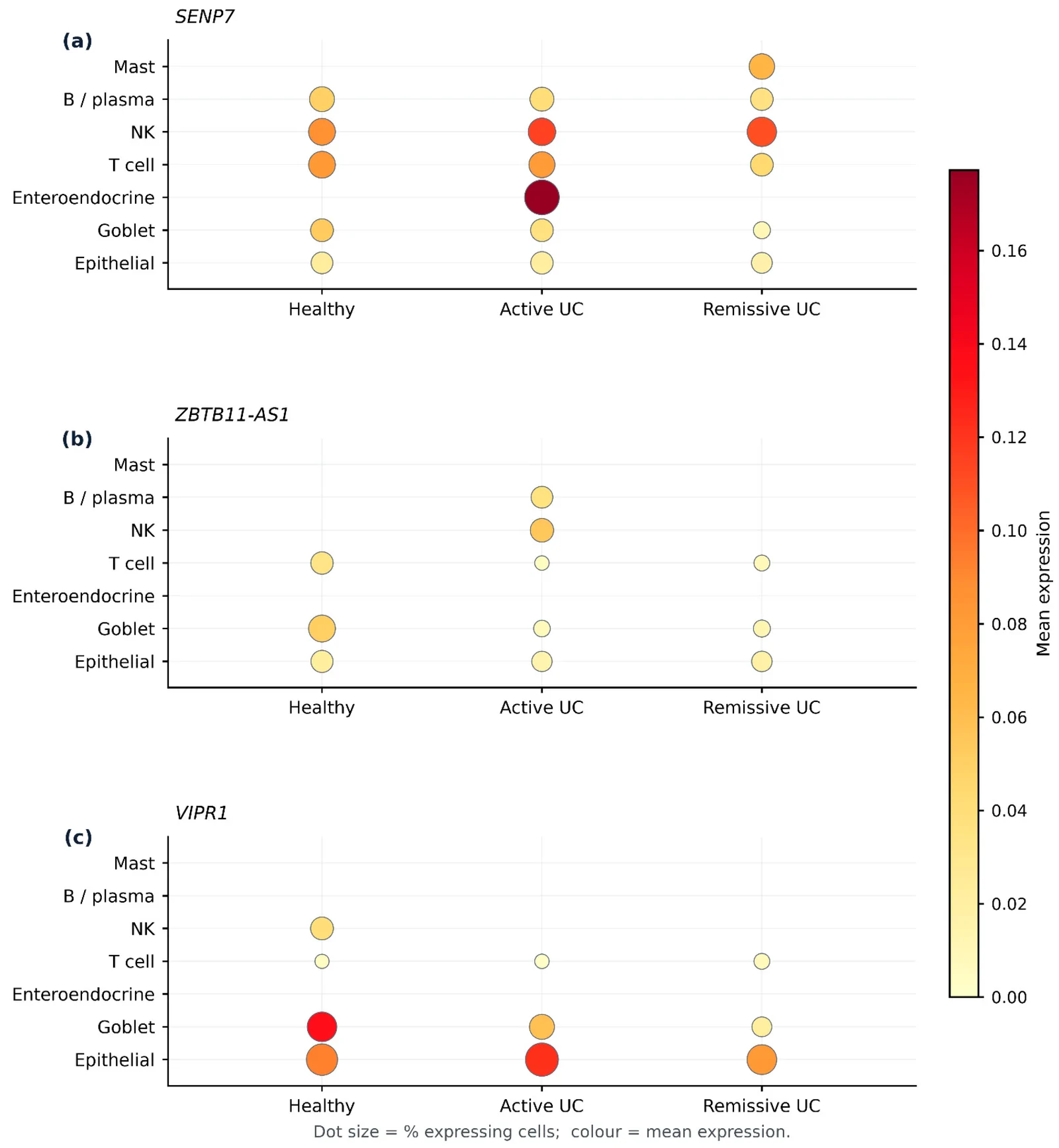
Single-cell expression of prioritized genes in colonic mucosa (GSE116222). (**a**–**c**) Dot/stacked-bar plots of the percentage of expressing cells and the mean expression of SENP7 (**a**), ZBTB11-AS1 (**b**) and VIPR1 (**c**) across epithelial, goblet, enteroendocrine, T/NK (T cell and NK), B/plasma and mast cell annotations in healthy controls, active UC and remissive UC (11,175 cells total; T/NK, B/plasma and mast cells are minor populations co-isolated with the epithelial fraction). Dot size indicates the percentage of expressing cells; colour indicates mean expression.

In 11,175 colonic mucosal cells, *SENP7* was detected at higher frequencies in T/NK cells (T: 6.2% health, 5.5% active, 3.1% remissive; NK: 6.3–9.1%) than in epithelial cells (2.1–2.9%), consistent with its whole-blood rather than colonic colocalization signal. *ZBTB11-AS1* was enriched in healthy goblet cells (6.25%) but collapsed in active (0.77%) and remissive (0.85%) UC. *VIPR1* was predominantly epithelial (11.7% health, 14.6% active, 9.4% remissive) and goblet (9.4% health → 4.6% active → 1.7% remissive), with negligible immune expression. Because cell annotation relied on a marker-panel heuristic, these fractions are interpreted as indicative localization rather than definitive quantification; the robust observations are the epithelial/goblet enrichment of *ZBTB11-AS1* and *VIPR1*, whereas the *SENP7* immune enrichment—based on small co-isolated T/NK populations—is supportive but indicative. Figure 5 summarizes the single-cell expression of SENP7, ZBTB11-AS1 and VIPR1 across cell types and disease states.

## 3. Discussion

The gut microbiome is extensively altered in IBD, yet separating causal microbial drivers from inflammation-induced dysbiosis has remained difficult. In this study, we integrated two-sample MR, independent population replication, cis-eQTL colocalization and single-cell mapping to address both the microbial and host-side questions. Our results identified several taxa with replicated genetically predicted associations and prioritized two host genes—*SENP7* (whole-blood eQTL; minor T/NK-cell detection) and *ZBTB11-AS1* in colonic epithelium—with distinct tissue logic, while also demonstrating that a substantial fraction of single-cohort MR candidates fail to survive replication or pleiotropy screening.

At the microbial level, our findings partly converge with earlier MR studies. Protective *Lactobacillus* → IBD (OR 0.78, directionally supported in FinnGen) aligns with recurring themes across several reports [13,14,15,16,17]. The risk-increasing *NB1n* lineage, by contrast, replicates more robustly than most previously reported taxa (OR 1.32 in IIBDGC, 1.60 in FinnGen) and fits the broader pattern of butyrate-producing *Lachnospiraceae*/*Ruminococcaceae* depletion in IBD [2,3,4,5]. However, we also downgraded several associations that had appeared significant in single-cohort analyses. *Catenibacterium* showed MR-Egger intercept and MR-PRESSO evidence of pleiotropy with direction reversal in FinnGen; the *E. hallii* group, despite nominal replication in FinnGen, lost significance after outlier correction in IIBDGC. These cases illustrate that MR significance alone is insufficient—replication and pleiotropy screening materially alter the candidate list and argue for caution when interpreting single-cohort reports.

The gene-level results provide the more novel contribution. *SENP7* encodes a deSUMOylase with context-dependent immune effects: in macrophages its knockdown suppresses NLRP3 inflammasome activation [20]; these findings sit within a broader gasdermin-D-driven pyroptotic programme implicated in mucosal inflammation [21,22,23], whereas intestinal epithelial *SENP7* loss ameliorates experimental colitis via γδ T-cell regulation [24]. Our blood expression-SMR points in the opposite direction of the epithelial model—higher whole-blood *SENP7* associates with lower CD risk (OR 0.70)—and aligns with the human genetic phenotype, where biallelic *SENP7* loss-of-function causes immunodeficiency with inflammatory features [25]. Importantly, colocalization indicates that the colonic *SENP7* eQTL and the IBD signal reflect two distinct causal variants (PP.H3 = 0.97 in sigmoid colon), whereas the whole-blood signal supports shared causality (PP.H4 = 0.86 for CD). Thus the intestinal epithelial and blood immune effects, while involving the same gene, are not the same causal signal. At the colonic locus, the relevant transcript is *ZBTB11-AS1*, a divergent-strand lncRNA whose expression colocalized with IBD (PP.H4 = 0.76) and CD (0.74) and tracked with higher disease risk (SMR OR 1.08–1.10). Single-cell data placed it in goblet cells in healthy mucosa, with marked loss in both active and remissive UC, implicating a mucus-lineage epithelial axis. lncRNAs are increasingly recognized as regulators of epithelial differentiation and barrier function [26], though *ZBTB11-AS1* itself remains functionally uncharacterized and merits direct experimental follow-up. A third locus, *VIPR1* (encoding the VPAC1 receptor for vasoactive intestinal peptide), is biologically attractive given VIP’s anti-inflammatory and barrier-supporting roles [27,28], but its upstream *Lachnospiraceae* UCG-001 association was direction-reversed in FinnGen, and the locus contained only 15 SNPs for colocalization. We therefore report it as a candidate for future investigation rather than as a finding of this study.

**Figure 6:**
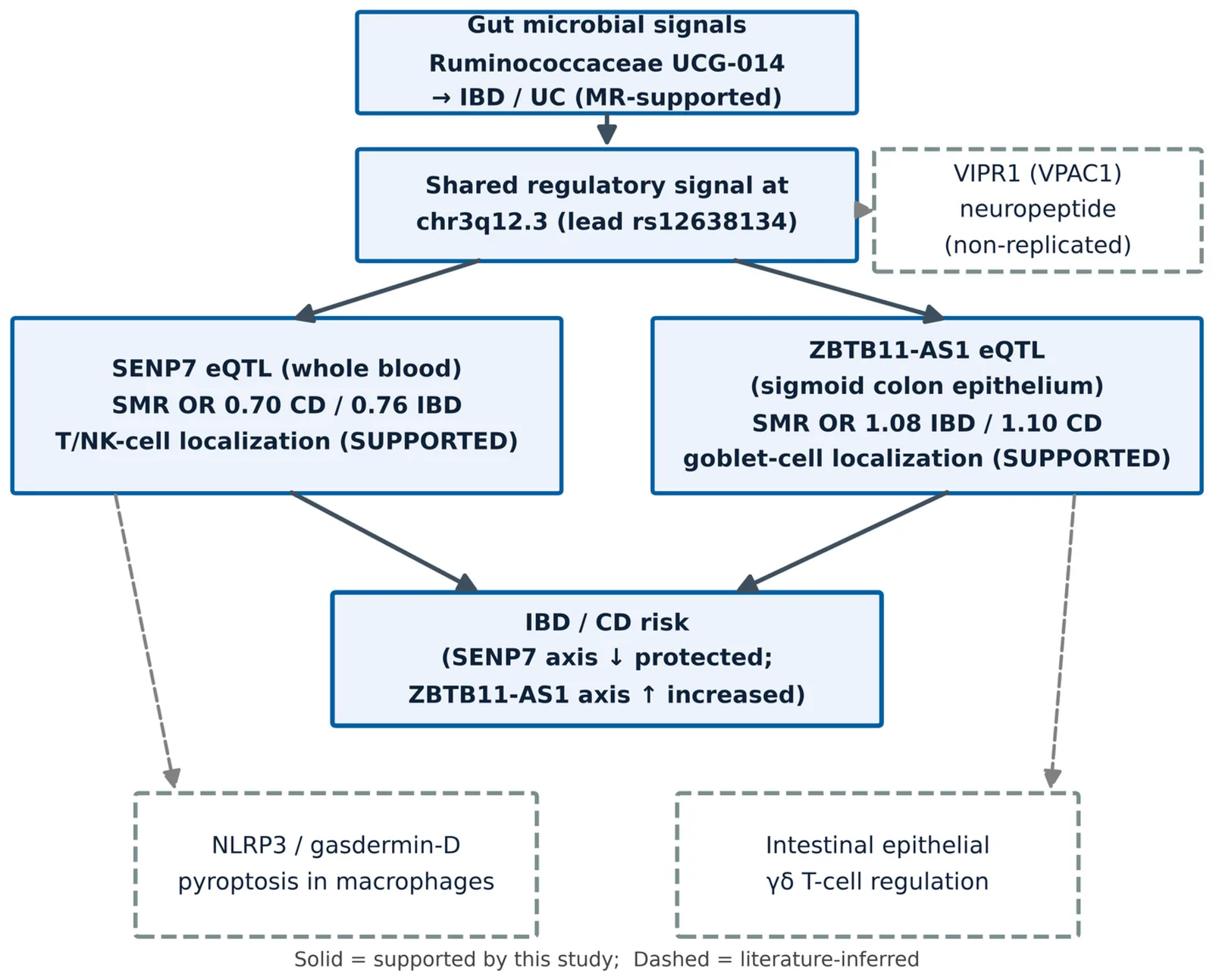
The proposed mechanistic model. An integrated, hypothesis-generating model linking the study’s supported associations. The solid lines denote pathways supported by the data in the present study: genetically influenced microbial signals converge on chr3q12.3 regulatory variants associated with SENP7 expression in whole blood and ZBTB11-AS1 expression in colonic epithelium. Single-cell detection of SENP7 in the minor T/NK populations captured within epithelial preparations and of ZBTB11-AS1 in epithelial/goblet cells is represented (SUPPORTED); the arrows from the eQTL boxes to IBD/CD risk denote colocalization/SMR-supported eQTL–disease associations (cis-SMR OR), not univariable MR estimates of gene expression on disease. The dashed lines denote literature-inferred biology not demonstrated here: NLRP3/gasdermin-D pyroptotic signalling in macrophages [20,21,22,23], epithelial gamma-delta T-cell regulation [24], and VIPR1 (VPAC1)-mediated neuropeptide signalling [27,28], the last arising from a non-replicated upstream taxon. The model is presented as a framework for experimental testing rather than as an established causal network.

The evidence is strongest for *SENP7*, supported by population-level replication, dual eQTL resources (GTEx and CAGE), formal colocalization, and single-cell localization. *ZBTB11-AS1* is supported at the population, cis-eQTL, supportive-colocalization and goblet-cell levels, but lacks functional characterization. *VIPR1* is supported only by eQTL and cell-type localization—consistent with its candidacy status. Grading the evidence in this way, rather than presenting a flat list, allows readers to weigh each gene on its own merits. Figure 6 presents the proposed mechanistic model linking these findings.

We are more cautious about UC-specific findings. *Ruminococcaceae UCG-014* → UC was directionally supportive but not statistically replicated in FinnGen; *ZBTB11-AS1* colocalization was weak for UC (PP.H4 = 0.10); and the *E. ventriosum* group signal, although strengthened by outlier removal, showed null replication. We therefore regard the UC-level conclusions as hypothesis-generating, with CD and combined-IBD evidence substantially stronger. Whether this asymmetry reflects genuine subtype-specific microbial effects or reduced power due to smaller UC GWAS sample sizes cannot be determined from our data.

Several limitations warrant emphasis. First, the exposure GWAS uses 16S rRNA sequencing, which resolves taxa mainly to genus and cannot capture strain-level functions or absolute abundance. Second, the MiBioGen exposure and IIBDGC outcome meta-analyses include partially overlapping European participants, and the relaxed instrument-selection threshold (*p* < 1 × 10^−5^) can increase the sensitivity of the MR estimates to such sample overlap, which may bias results towards the confounded observational direction; although two-sample MR estimates remain valid under modest overlap, residual overlap-driven bias is retained as a limitation. All participants were of predominantly European ancestry, which limits generalizability, and LD clumping was performed against the 1000 Genomes European reference panel; therefore, for the minority of variants affected by population substructure the LD matching may be imperfect (expected effect modest). Third, formal multivariable MR to separate correlated taxa was not feasible: because MiBioGen releases only *p* < 1 × 10^−4^ hits, all 84 pairwise taxon combinations had zero shared instrumental SNPs, so residual correlated pleiotropy between taxa cannot be fully excluded [29]. Fourth, reverse-direction MR (IBD → microbiota) was not estimable for the same reason—the public MiBioGen release lacks genome-wide summary statistics, so disease-associated instruments generally lack microbiota effect estimates. Fifth, eQTL–disease colocalization tested sharing between the cis-eQTL and the IBD GWAS signal rather than between the microbiota GWAS and the eQTL, so the microbiota-to-gene link relies on the shared instrument SNP at each locus; it also remains sensitive to the prior probability of a shared causal variant (PP.H4 values supporting colocalization under the default p12 = 1 × 10^−5^ attenuated under the stricter p12 = 1 × 10^−6^), so we interpret colocalization as supportive triangulation rather than definitive localization, alongside SMR and cross-cohort consistency. Sixth, all MR and colocalization computations used a custom Python implementation that was not independently benchmarked against reference software; the approximated Steiger test inferred directionality from *p* values and sample sizes rather than individual-level data, and HEIDI was not performed because matched full cis-eQTL summaries with LD were unavailable. Seventh, single-cell annotation used a marker-panel heuristic without reference mapping, and the enteroendocrine subset contained only 11 cells; cell-type percentages should be treated as indicative. Eighth, weak-instrument and winner’s-curse biases remain possible despite F > 10 filtering, and some instruments were small (3–6 SNPs), making estimates for taxa such as *Catenibacterium* particularly fragile.

## 4. Materials and Methods

### 4.1. Study Design and Assumptions

We conducted a two-sample summary-data MR study following the STROBE-MR reporting guideline (checklist provided as supplementary material) [30]. MR rests on three assumptions: IVs are (1) strongly associated with exposure, (2) independent of confounders of the exposure–outcome relationship, and (3) associated with the outcome only through the exposure (no horizontal pleiotropy) [10,11]. We addressed assumption 1 with F-statistics, assumption 2 through the random allocation of genotype, and assumption 3 using an array of pleiotropy-robust estimators, colocalization and directionality tests described below. All analyses used a custom Python implementation (Python 3, NumPy/SciPy/pandas) built directly from the published statistical formulations; no third-party (Python 3.9; NumPy>=1.21, SciPy>=1.7, pandas>=1.3, statsmodels>=0.13, per the requirements of the public code repository)MR software package was invoked.

### 4.2. GWAS Data Sources

Microbial exposure GWAS were obtained from MiBioGen (24 cohorts, 18,340 individuals, predominantly European ancestry ~13,266; 16S rRNA gene sequencing), which provides genome-wide association summary statistics for 211 bacterial taxa spanning phylum to genus [12]. Outcome GWAS were the IIBDGC European-ancestry meta-analyses of de Lange and colleagues: IBD (GCST004131; 25,042 cases/34,915 controls), CD (GCST004132; 12,194/28,072) and UC (GCST004133; 12,366/33,609), provided in NCBI build 38 (GRCh38/hg38) coordinates [9]. Independent replication used FinnGen release R12 (IBD 10,960 cases/500,348 controls; CD 2489/500,111; UC 7220/499,380) [31]. Genomic builds differ across datasets: the IIBDGC outcome summary statistics and the regional plots in Figure 4 are presented on GRCh38 (hg38), whereas the MiBioGen exposure and GTEx v8 eQTL datasets use GRCh37 (hg19). Because all variant harmonization in this study was performed by rsID and effect/other allele matching (a position-independent procedure), this build difference did not affect the MR or colocalization variant matching, and no cross-build coordinate conversion was required for the variant-alignment step; as displayed in Figure 4, the GRCh37-based GTEx v8 eQTL variants were lifted to hg38 coordinates (pos_hg38 = pos_hg19 + 281,156 bp, the verified offset for the SENP7 locus) to share a common axis with the natively hg38 GWAS; under this conversion the three lead SNPs (rs7631506, rs12638134 and rs2081577) aligned exactly between datasets, and because variant matching remained rsID- and allele-based, this display-level conversion did not affect any posterior probability. Strand and allele alignment between the eQTL and GWAS datasets was verified for every variant entering a colocalization window, and palindromic (A/T, C/G) variants were excluded from cross-dataset alignment to avoid strand ambiguity. All datasets were derived from publicly available, de-identified summary statistics; no new ethical approval was required. The complete list of GWAS and functional data sources is summarized in Table 3.

### 4.3. Instrumental Variable Selection

For each taxon, independent single-nucleotide polymorphisms (SNPs) associated at *p* < 1 × 10^−5^ were selected as candidate IVs. This relaxed threshold (rather than genome-wide *p* < 5 × 10^−8^) was chosen because microbiota traits are highly polygenic and 16S-based taxon associations are individually weak: at the conventional threshold most taxa would have yielded zero or one instrument, making multi-SNP estimators inapplicable. The threshold is standard in published microbiota MR [13,14,15,16,17], and its lower per-SNP strength was compensated by stringent downstream filtering—LD pruning, F-statistic exclusion, pleiotropy-robust estimators, outlier correction and independent replication—so that only signals surviving multiple screens were interpreted. Linkage disequilibrium (LD) clumping was performed in PLINK 1.9 using the 1000 Genomes Project European reference panel (503 unrelated EUR individuals), retaining the strongest association per clump defined by a 10 Mb physical window at r^2^ < 0.001 [34,36]. The stringent r^2^ and window settings minimize residual correlation between instruments, which matters because the IVW estimator assumes independent SNPs. Palindromic variants (A/T and C/G) and any strand-ambiguous alleles were excluded to avoid mis-orientation, and exposure and outcome effect alleles were manually cross-checked for every retained SNP; outcome effect estimates were flipped to the exposure effect-allele orientation where the reference alleles matched. Instrument strength was quantified per SNP with F = β^2^/SE^2^ using the exposure association statistics (mean and minimum F reported per taxon); variants with F < 10, the conventional weak-instrument threshold, were excluded. No phenotype-scan-based filtering (e.g., lookup in external trait databases) was applied; instrument selection used only the microbiota GWAS. Taxa quantified at multiple Linnaean levels that shared an identical clumped SNP set (the unclassified NB1n lineage reported at order, family and genus) were retained in the 633-test framework but counted as one independent feature for interpretation. The MiBioGen public release provides only *p* < 1 × 10^−4^ association hits rather than genome-wide summary statistics, which precludes construction of union instrument sets for joint multi-exposure models (see Section 3).

### 4.4. Mendelian Randomization Analyses

The primary estimator was the inverse-variance weighted (IVW) method, implemented as the weighted regression of SNP-outcome on SNP-exposure effects [37]. Because instrument-level heterogeneity inflates fixed-effect IVW standard errors, we applied a pre-specified dual-reporting rule: for any signal with nominally significant heterogeneity (Cochran’s Q *p* < 0.05), the IVW result was reported alongside a multiplicative random-effects IVW estimate, whose standard error is inflated by the over-dispersion factor sqrt(Q/df) (df = number of instruments minus one), and the MR-PRESSO outlier-corrected estimate. Signals were then classified by a fixed decision tree: (i) retained as primary associations when both the random-effects IVW and the MR-PRESSO-corrected estimate remained significant (*p* < 0.05); (ii) retained but flagged as presso-robust/RE-sensitive when only the MR-PRESSO-corrected estimate remained significant; and (iii) downgraded to suggestive when neither remained significant. This rule was defined a priori, applied uniformly to all 15 FDR-significant signals, and was not adjusted based on individual results, to preclude selective reporting. MR estimates were generated for all 633 taxon–outcome combinations (211 taxa × 3 outcomes). Odds ratios (ORs) represent the change in IBD odds per 1-standard-deviation increase in genetically predicted taxon relative abundance. In the MiBioGen pipeline, non-zero taxon relative-abundance counts were natural log-transformed, regressed on study-specific covariates, and the residuals were scaled and centred to mean 0 and SD 1 before association testing; each SNP–taxon effect is therefore expressed per allele × SD of the log-transformed, covariate-adjusted relative-abundance trait, and we preserved this scale unchanged in our harmonization [12]. Complementary estimators were MR-Egger regression (which allows directional pleiotropy via a non-zero intercept) [18] and the weighted-median estimator (consistent if ≥50% of the weight comes from valid instruments) [38]. Heterogeneity was assessed with Cochran’s Q. We specify the minimum instrument number under which each sensitivity estimator is meaningful and restrict the interpretive weight assigned to these statistics accordingly: the weighted-median estimator is valid with ≥3 instruments; MR-Egger requires ≥3 instruments to define an intercept but its slope and intercept are numerically stable only with larger panels; and MR-PRESSO’s global test and outlier detection require more instruments than variants so that the residual-sum-of-squares null distribution is estimable (practically ≥4 robust instruments). Consequently, for instrument sets with fewer than five SNPs, MR-Egger intercept and MR-PRESSO outlier statistics are reported for completeness only and are not assigned inferential weight equal to analyses based on larger instrument sets. Horizontal pleiotropy was evaluated with MR-PRESSO, using a Python implementation following Verbancket al. (global test, outlier test and outlier-corrected estimate; 1000 simulations per signal, random seed 20240830) [19]. Leave-one-out analyses iteratively excluded each SNP. Directionality was examined with an approximated Steiger directionality test: because per-SNP explained variance was not supplied, SNP-exposure and SNP-outcome R^2^ were back-calculated from reported *p* values and sample sizes, and the Steiger Z statistic compared the variance explained on each side [39]. Finally, to cross-validate the custom pipeline against established software, we independently recomputed all 15 FDR-significant IVW signals with the R TwoSampleMR package (version 0.5.6) (using its standard harmonise_data() workflow, including allele harmonization, strand flipping and removal of palindromic variants) on the raw exposure–outcome summary statistics. The TwoSampleMR IVW point estimates (beta and odds ratio) matched our custom Python estimates for all 15/15 signals exactly (differences < 1 × 10^−9^), confirming that the effect directions and magnitudes are robust and independent of any single implementation. Consistent with our dual-reporting rule above, the only difference between the two implementations was in the standard errors: for signals with nominally significant heterogeneity (Cochran’s Q *p* < 0.05), TwoSampleMR internally switches to a multiplicative random-effects IVW, whereas our pipeline reports both the fixed-effect IVW and the random-effects IVW whose standard error is inflated by the over-dispersion factor √(Q/df), together with the MR-PRESSO outlier-corrected estimate, and classifies signals according to the pre-specified rule; the point estimates agree exactly. The TwoSampleMR benchmarking script, its JSON input files, and the comparison report are provided in the public code repository. The complete code, benchmarking scripts, input files, and reproducibility workflow are available at https://github.com/slkc2020/ibd-microbiota-mr (accessed on 29 August 2026).

### 4.5. Replication

The 15 FDR-significant discovery signals were carried forward for replication in FinnGen R12, irrespective of their subsequent heterogeneity classification. Replication was defined a priori as nominal *p* < 0.05 with concordant effect direction; direction-concordant but non-significant associations were considered supportive. No additional multiple-testing correction was applied to the replication *p* values: the 15 candidate signals formed a small, pre-specified set fixed by the discovery phase, in which FDR control was the primary inferential step, and nominal *p* < 0.05 with concordant direction is the standard, transparent convention in two-sample MR replication studies; the replicated fraction is reported transparently so that it can be compared with chance expectation.

### 4.6. Multiple Testing

Benjamini–Hochberg false discovery rate (FDR) correction was applied separately within each outcome across the 211 taxon tests [40]. FDR < 0.05 defined statistical significance. As a stricter sensitivity analysis, FDR was also computed across all 633 tests jointly.

### 4.7. cis-eQTL Summary-Data MR and Colocalization

To connect microbiota-associated loci to host genes, lead microbiota IV SNPs were inspected for cis-regulatory potential: all genes within a 1 Mb window were queried, and where a lead SNP showed a cis-eQTL association (*p* < 1 × 10^−5^) for a gene in a disease-relevant tissue, that gene was carried forward. cis-eQTL evidence was extracted from GTEx v8 in whole blood (n = 670), sigmoid colon (n = 369), transverse colon (n = 369) and terminal ileum (n = 173) [32], chosen to represent the hematopoietic immune compartment and the gut epithelial compartment respectively; whole-blood eQTL effects were independently checked in the CAGE consortium blood eQTL meta-analysis (n = 2765); cross-consortium replication was assessed at the gene level (same gene, concordant effect direction), since the lead variants differ between datasets [33]. Two complementary analyses were then performed. (1) cis-SMR: the association between genetically predicted gene expression and IBD was estimated as the Wald ratio of the lead/top cis-eQTL SNP’s disease effect to its expression effect, which tests whether the expression trait mediates the locus–disease association [41]. A multi-SNP IVW-SMR across all cis-eQTL SNPs in the window was also computed but treated as archive only, because without the local LD matrix its standard errors are anti-conservative; all reported SMR inferences therefore use the single top-cis-eQTL Wald ratio. (2) Bayesian colocalization with coloc.abf (version 5.2.2), which compares five hypotheses (no association, association in one trait only, or shared versus distinct causal variants) using approximate Bayes factors [42]. We used the software’s default priors (p1 = p2 = 1 × 10^−4^ for a SNP associated with either trait; p12 = 1 × 10^−5^ for association with both) and Wakefield approximate Bayes factors with prior effect-size variances W1 = 0.04^2^ for the quantitative expression trait and W2 = 0.15^2^ for binary disease [43,44]. The posterior probability of a single shared causal variant (PP.H4) > 0.80 was considered strong colocalization, 0.50–0.80 supportive, while PP.H3 (two distinct causal variants) > 0.80 indicated that the eQTL and disease signals, though colocalizing geographically, arise from different variants. The primary analysis used the coloc.abf default prior p12 = 1 × 10^−5^ [42]; because the posterior for a shared causal variant is sensitive to this prior, a stricter sensitivity analysis with p12 = 1 × 10^−6^ was also run and is reported alongside. Loci with fewer than 50 SNPs in the colocalization window were deemed uninformative and not tested. The SMR HEIDI test, which distinguishes linkage from pleiotropy using full cis-eQTL summary statistics matched to an LD reference, was not performed because the complete per-variant eQTL statistics with compatible LD matrices were not available to us; we therefore assessed pleiotropy at gene loci through formal colocalization and cross-cohort direction consistency, and note this limitation explicitly.

### 4.8. Single-Cell Localization and Software

Expression of prioritized genes was examined in GSE116222, 10× Chromium single-cell RNA-seq of colonic epithelial isolations from healthy controls and patients with active or remissive UC (11,175 cells after quality control) [35]; the dataset is an epithelial-cell census, so annotated T/NK, B/plasma and mast cells represent minor mucosal immune populations co-isolated with the epithelial fraction, and immune-cell detection frequencies are therefore indicative only. Cells were annotated with a marker-panel heuristic using 13 canonical marker panels (Epithelial: EPCAM/KRT8/KRT18/KRT19; Goblet: MUC2/TFF3/FCGBP/ZG16; Tuft: DCLK1/TRPM5/POU2F3; Enteroendocrine: CHGA/CHGB/NEUROD1; Stem: LGR5/OLFM4/SMOC2/MKI67; T cell: CD3D/CD3E/CD2/TRAC; NK: NKG7/GNLY/GZMB/KLRF1; B/plasma: MS4A1/CD79A/MZB1/JCHAIN/IGHG1; Macrophage: CD68/CD163/LST1/C1QA/S100A8; Dendritic cell: FCER1A/CLEC10A/ITGAX/HLA-DQA1; Mast: TPSAB1/CPA3/KIT/HDC; Fibroblast: COL1A1/COL1A2/DCN/COL3A1/LUM; Endothelial: PECAM1/VWF/CLDN5/CDH5/EMCN). For each cell, a per-panel score was computed as the proportion of markers in that panel with detectable expression, and the cell was assigned to the highest-scoring panel; cells whose top-panel score fell below 0.34 (i.e., on average fewer than about one canonical marker confidently expressed) were assigned to ‘Unknown’ to avoid spurious annotation; no external reference mapping was used. Counts were library-size-normalized and log-transformed, with cells retained by standard single-cell quality control; the complete marker panel, scoring code and QC details are deposited in the repository, and the percentage of expressing cells and mean expression were tabulated by cell type and disease state. Data handling used Python (NumPy, SciPy, pandas, statsmodels); PLINK 1.9 was used for LD clumping only.

## 5. Conclusions

In conclusion, this study provides replicated evidence for genetically predicted microbial associations with IBD and prioritizes *SENP7* and *ZBTB11-AS1* as host genes with distinct tissue-specific signatures, while demonstrating that replication and pleiotropy screening materially change the candidate landscape. The prioritized genes and microbial features offer a testable shortlist for mechanistic follow-up and, if corroborated, may inform host-directed or microbiome-targeted strategies in IBD.

## Figures and Tables

**Table 1:** FDR-significant microbiota–IBD associations in IIBDGC and FinnGen replication.

Outcome	Microbial Feature	n SNP	β (SE)	OR (95% CI)	*p*	FDR	Classification	Q *p*	IIBDGC Sensitivity	FinnGen OR (95% CI)	FinnGen *p*	Direction	Replication
CD	NB1n order (unclassified Clostridia)	6	0.277 (0.070)	1.32 (1.15–1.51)	8.0 × 10^−5^	0.006	Main	0.10	PRESSO ns; LOO stable	1.60 (1.24–2.07)	2.4 × 10^−4^	Same	Replicated
CD	*Catenibacterium*	3	−0.286 (0.079)	0.75 (0.64–0.88)	3.1 × 10^−4^	0.017	Suggestive	4.1 × 10^−4^	Egger intercept *p* = 3.2 × 10^−11^; PRESSO fragile (2/3 outliers)	1.23 (0.93–1.64)	0.151	Reversed	Not replicated
CD	*Lachnospiraceae UCG-001*	10	−0.258 (0.076)	0.77 (0.67–0.90)	6.8 × 10^−4^	0.029	Main	0.42	PRESSO ns	1.12 (0.87–1.44)	0.392	Reversed	Not replicated
CD	*Eubacterium hallii* group	8	−0.359 (0.110)	0.70 (0.56–0.87)	1.1 × 10^−3^	0.038	Suggestive	5.5 × 10^−6^	PRESSO: *p* = 0.241 after outlier (sig → ns)	0.69 (0.50–0.94)	0.018	Same	Replicated †
CD	*Ruminococcaceae UCG-009*	7	−0.265 (0.083)	0.77 (0.65–0.90)	1.4 × 10^−3^	0.042	Main	0.58	PRESSO ns	1.01 (0.76–1.35)	0.930	Reversed	Not replicated
IBD	*Ruminococcaceae UCG-014*	6	0.352 (0.092)	1.42 (1.19–1.70)	1.2 × 10^−4^	0.020	Main	0.091	PRESSO ns (0.086); LOO stable	1.13 (0.91–1.39)	0.271	Same	Direction-supportive
IBD	NB1n order	6	0.192 (0.054)	1.21 (1.09–1.35)	3.8 × 10^−4^	0.020	PRESSO-robust/RE-sensitive	0.0045	PRESSO *p* = 0.014 after outliers (sig)	1.14 (1.01–1.28)	0.040	Same	Replicated
IBD	*Catenibacterium*	3	−0.208 (0.062)	0.81 (0.72–0.92)	7.7 × 10^−4^	0.027	Main	0.079	Egger intercept *p* = 6.1 × 10^−42^	1.02 (0.88–1.17)	0.811	Reversed	Not replicated
IBD	*Lactobacillus*	5	−0.245 (0.073)	0.78 (0.68–0.90)	7.7 × 10^−4^	0.027	Main	0.90	PRESSO ns (0.913)	0.90 (0.80–1.01)	0.071	Same	Direction-supportive
UC	*Eubacterium ventriosum* group	10	−0.377 (0.098)	0.69 (0.57–0.83)	1.2 × 10^−4^	0.025	Main	0.027	PRESSO *p* = 1.7 × 10^−6^ after outlier (stronger)	0.98 (0.81–1.18)	0.833	Same	Not replicated
UC	*Ruminococcaceae UCG-014*	6	0.417 (0.117)	1.52 (1.21–1.91)	3.5 × 10^−4^	0.037	Main	0.15	PRESSO ns (0.162)	1.17 (0.90–1.52)	0.238	Same	Direction-supportive

Signals were classified based on Cochran’s Q and MR-PRESSO: Main (no significant heterogeneity, or significant under both random-effects IVW and MR-PRESSO after outlier correction), PRESSO-robust/RE-sensitive (not significant under random-effects IVW but significant after MR-PRESSO outlier correction), and suggestive (not significant under either estimator, or MR-PRESSO unreliable with too few SNPs). Random-effects IVW standard errors were inflated by the over-dispersion factor √(Q/df). † Replication was defined a priori as nominal *p* < 0.05 with concordant effect direction; *E. hallii* → CD replicated in FinnGen despite being downgraded to suggestive.

**Table 2:** cis-eQTL, SMR and colocalization evidence for prioritized genes.

Gene	Tissue	Lead SNP (cis-eQTL β, *p*)	Outcome	coloc PP.H4 (PP.H3), n SNP	cis-SMR Wald OR (*p*)	Single-Cell Localization
*SENP7*	Whole blood (GTEx; CAGE replicated)	rs12638134 → β −0.126, 4.2 × 10^−14^ (top rs7631506 1.2 × 10^−32^)	CD	0.857 (0.141), 590	0.698 (1.7 × 10^−5^)	NK 6.3–9.1%; T cells 3.1–6.2%; epithelial ~2–3%
*SENP7*	Whole blood	—	IBD	0.695 (0.304), 590	0.757 (1.8 × 10^−5^)	as above
*SENP7*	Whole blood	—	UC	0.319 (0.064), 590	0.814 (0.012)	as above
*SENP7*	Sigmoid colon	rs12638134 → β −0.183, 1.2 × 10^−7^	IBD	0.0007 (0.973), 532	0.960 (0.32)	low epithelial
*ZBTB11-AS1*	Sigmoid colon	rs12638134 → β +0.385, 6.1 × 10^−8^ (top rs2081577 4.2 × 10^−23^)	IBD	0.761 (0.145), 287	1.076 (1.4 × 10^−4^)	goblet 6.25% health → 0.8% UC; epithelial ~2%
*ZBTB11-AS1*	Sigmoid colon	—	CD	0.740 (0.230), 287	1.104 (7.0 × 10^−5^)	as above
*ZBTB11-AS1*	Sigmoid colon	—	UC	0.102 (0.015), 284	1.058 (0.021)	as above
*VIPR1* (from UCG-001 instrument; upstream taxon not replicated)	Whole blood (GTEx; CAGE *p* = 7.7 × 10^−78^)	rs437876 → β +0.135, 1.4 × 10^−7^	IBD/CD/UC	not estimable (15 SNP)	0.91–1.03 (0.38–0.80)	epithelial 11.7–14.6%; goblet 9.4% → 1.7%

**Table 3:** Summary of GWAS and functional data sources.

Dataset	Role	Sample Size	Accession/Reference
MiBioGen (24 cohorts, 16S rRNA)	Exposure: 211 microbial taxa	18,340 individuals (~13,266 European)	Kurilshikov et al., Nat Genet 2021 [12]
IIBDGC (de Lange 2017) [9]	Outcome: IBD/CD/UC	IBD 25,042/34,915; CD 12,194/28,072; UC 12,366/33,609 (cases/controls)	GWAS Catalog GCST004131/32/33 [9]
FinnGen R12	Independent replication	IBD 10,960/500,348; CD 2489/500,111; UC 7220/499,380 (cases/controls)	Kurki et al., Nature 2023 [31]
GTEx v8	cis-eQTL (blood, ileum, colon)	Whole blood 670; sigmoid colon 369; transverse colon 369; terminal ileum 173	GTEx Consortium, Science 2020 [32]
CAGE	Whole-blood cis-eQTL replication	2765	Lloyd-Jones et al., 2017 [33]
1000 Genomes EUR	LD reference for clumping	503	1000 Genomes Project, Nature 2015 [34]
GSE116222	Single-cell colonic epithelium	11,175 cells from epithelial isolations (healthy/active UC/remissive UC; minor co-isolated immune populations)	Parikh et al., Nature 2019 [35]

## Data Availability

All datasets analyzed in this study are publicly available and can be accessed as follows: MiBioGen gut microbiota GWAS summary statistics (https://mibiogen.gcc.rug.nl accessed on 29 August 2026); IIBDGC GWAS summary statistics via the GWAS Catalog under accession GCST004131, GCST004132 and GCST004133; FinnGen release R12 summary statistics (https://r12.finngen.fi/ accessed on 29 August 2026); GTEx v8 eQTL data (https://gtexportal.org accessed on 29 August 2026); CAGE eQTL data via the Center for Neurogenomics and Cognitive Research (https://cnsgenomics.com/data accessed on 29 August 2026); and single-cell RNA-sequencing data in the Gene Expression Omnibus under accession GSE116222. The custom Python analysis code, the R TwoSampleMR benchmarking scripts, their input files, software versions, dependencies, and random seeds, together with a reproducible end-to-end workflow, are deposited in the public code repository accompanying this manuscript: https://github.com/slkc2020/ibd-microbiota-mr (accessed on 29 August 2026).

## Supplementary Materials

The following supporting information can be downloaded at https://www.mdpi.com/article/10.3390/ijms27198718/s1.
