# Supplementary material for: Gut Microbiota, Inflammatory Bowel Disease and the Prioritization of Host Effector Genes: A Two-Sample Mendelian Randomization Study with Independent Replication, cis-eQTL Colocalization and Single-Cell Localization
Source: Int J Mol Sci. 2026 Sep 29;27(19):8718. doi: 10.3390/ijms27198718 (PMC13665393; doi:10.3390/ijms27198718)
Supplement: Supplementary file 1 [file ijms-27-08718-s001.zip › ijms-4581282-supplementary.pdf]

STROBE-MR Checklist (v2021)

Manuscript: Gut microbiota, inflammatory bowel disease and the prioritization of host effector genes: a two-sample Mendelian randomization study with independent replication, cis-eQTL colocalization and single-cell localization **Version:** manuscript_draft_v1.9.md **STROBE-MR reference:** Skrivankova VW, et al. JAMA. 2021;326(16):1614–21 (ref. [18])

#	Item	Recommendation	Location in manuscript
**1**	**Title and abstract**	Indicate that the study used Mendelian randomization (MR) in the title.	Title: "…: a two-sample Mendelian randomization study with independent replication, cis-eQTL colocalization and single-cell localization"
**2**	—	Provide structured abstract: background, methods, results, conclusions; state use of MR.	Abstract (pp. 14–27): four-part structured abstract (Background/Methods/Results/Conclusions); MR methods stated explicitly.
—	**Introduction**		
**3**	**Background and rationale**	Explain the scientific background and rationale for the MR study.	Background (pp. 28–37): rationale for MR (dysbiosis vs. reverse causation; observational confounds); gap (single-cohort, taxon-level, no host-gene link).
**4**	**Objectives**	State the specific objectives, including pre-specified causal hypotheses.	Background final paragraph: "Our aim was to prioritize candidate host genes through which microbiota-associated variants may influence IBD risk."
—	**Methods**		
**5**	**Study design and data sources**	Present key elements of study design. State the data sources, numbers of participants, and version numbers.	Methods → GWAS data sources (pp. 44–47): MiBioGen (211 taxa, 18,340 individuals); IIBDGC de Lange 2017 (IBD 25,042/34,915; CD 12,194/28,072; UC 12,366/33,609); FinnGen R12 (10,960 IBD cases / 500,348 controls; CD 2,489 / 500,111; UC 7,220 / 499,380).
**6**	**Assumptions**	Describe the three core MR assumptions and how they were assessed.	Methods → Study design and assumptions (pp. 40–43): IV relevance (F-statistics), independence (random allocation), exclusion restriction (pleiotropy-robust estimators, colocalization, directionality).
**7**	**Statistic**	Describe: (a) primary	Methods → MR analyses (pp. 52–55): IVW

	**al method s**	estimator; (b) how data were harmonized; (c) sensitivity analyses.	primary; harmonization (exclusion of palindromic/strand-ambiguous variants, effect-allele flips); sensitivity (Egger, weighted median, Cochran Q, MR-PRESSO, leave-one-out, approximated Steiger).
**8**	—	Describe how confounding and selection bias were addressed.	Methods → Study design and assumptions; Instrument construction (pp. 40–49): random allocation of genotype; exclusion based on F < 10; no phenotype-scan-based selection.
**9**	—	Describe whether and how analyses accounting for multiple testing were done (e.g. FDR).	Methods → Multiple testing (pp. 60–63): Benjamini–Hochberg FDR within each outcome; joint correction across all 633 tests as sensitivity.
**10**	—	Describe methods used for gene prioritization / follow-up of instruments (if applicable).	Methods → cis-eQTL summary-data MR and colocalization (pp. 64–67): cis-SMR Wald ratio, coloc.abf with dual-prior sensitivity; single-cell localization (pp. 68–73).
**11**	—	Describe how instruments and data were obtained and validated (software and datasets).	Methods → Instrumental variable selection (pp. 48–51): LD clumping in PLINK 1.9 (1000 Genomes EUR); F > 10 filtering. Software: Python 3 (NumPy, SciPy, pandas, statsmodels).
**12**	**Descrip tive data**	Report numbers of included participants/excluded; show characteristics.	Results → Instrument construction (pp. 76–79): 1,278 SNP–trait instruments; 197 taxa ≥3 IVs (median F ≈ 21), nine with two, five with one; all F > 10; 633 taxon–outcome tests.
—	**Results**		
**13**	**Main results**	Report the estimates and precision (OR, 95% CI), the effect size (per-SD), and P values.	Results → Main MR associations (pp. 80–89) + Table 2. All ORs are per 1-SD; 95% CIs and FDR-P reported.
**14**	**Sensitiv ity analyse s**	Report results of sensitivity analyses (Egger, weighted median, MR-PRESSO, leave-one-out, Steiger).	Results → Sensitivity analyses and replication (pp. 90–95); pleiotropy-robust estimator concordance stated.
**15**	**Replicat ion / additio**	Report results of independent replication and any additional	Results → Sensitivity analyses and replication (FinnGen R12, 7 replicated / 11 direction-concordant); Gene

	**nal analyses**	analyses.	prioritization and Single-cell localization.
—	**Discussion**		
16	**Key results**	Summarize key findings with reference to causal hypotheses.	Discussion (pp. 110–125): prioritized SENP7 and ZBTB11-AS1 with distinct tissue logic; taxon-level replication results.
17	**Limitations**	Discuss limitations; address sources of potential bias, multiplicity, IV assumptions violation.	Discussion → Limitations (8 numbered points): 16S resolution; ancestry; no MVMR (correlated pleiotropy); reverse MR not estimable; colocalization prior sensitivity; HEIDI not performed; single-cell heuristic annotation; weak-instrument/winner's-curse.
18	**Interpretation**	Give cautious overall interpretation consistent with limitations; compare with other evidence.	Discussion: evidence-grading (SENP7 strongest; ZBTB11-AS1 supportive; VIPR1 candidate); cautious UC interpretation; comparison with prior microbiota-MR and functional literature.
19	**Generalisability**	Discuss generalisability to other populations/contexts.	Discussion → Limitations #2 (European ancestry) and #7; Results on UC/CD asymmetry.
—	**Other information**		
20	**Funding**	State funding source and role of funders.	Declarations → Funding (Guangxi NSF 2022GXNSFAA035453; NSFC 22267002; GYJK 2023003; Z-A20221275).

STROBE-MR (Strengthening the Reporting of Observational Studies in Epidemiology Using Mendelian Randomization) — 10-item checklist with 20 sub-items. Completed in accordance with Skrivankova et al., JAMA 2021;326(16):1614–1621 (ref. [18]).
